# Supplementary material for: Sleeping Beauty transposon mutagenesis identified genes and pathways involved in inflammation-associated colon tumor development
Source: Nat Commun. 2023 Oct 16;14:6514. doi: 10.1038/s41467-023-42228-z (PMC10579371; doi:10.1038/s41467-023-42228-z)

Supplementary Figure 1

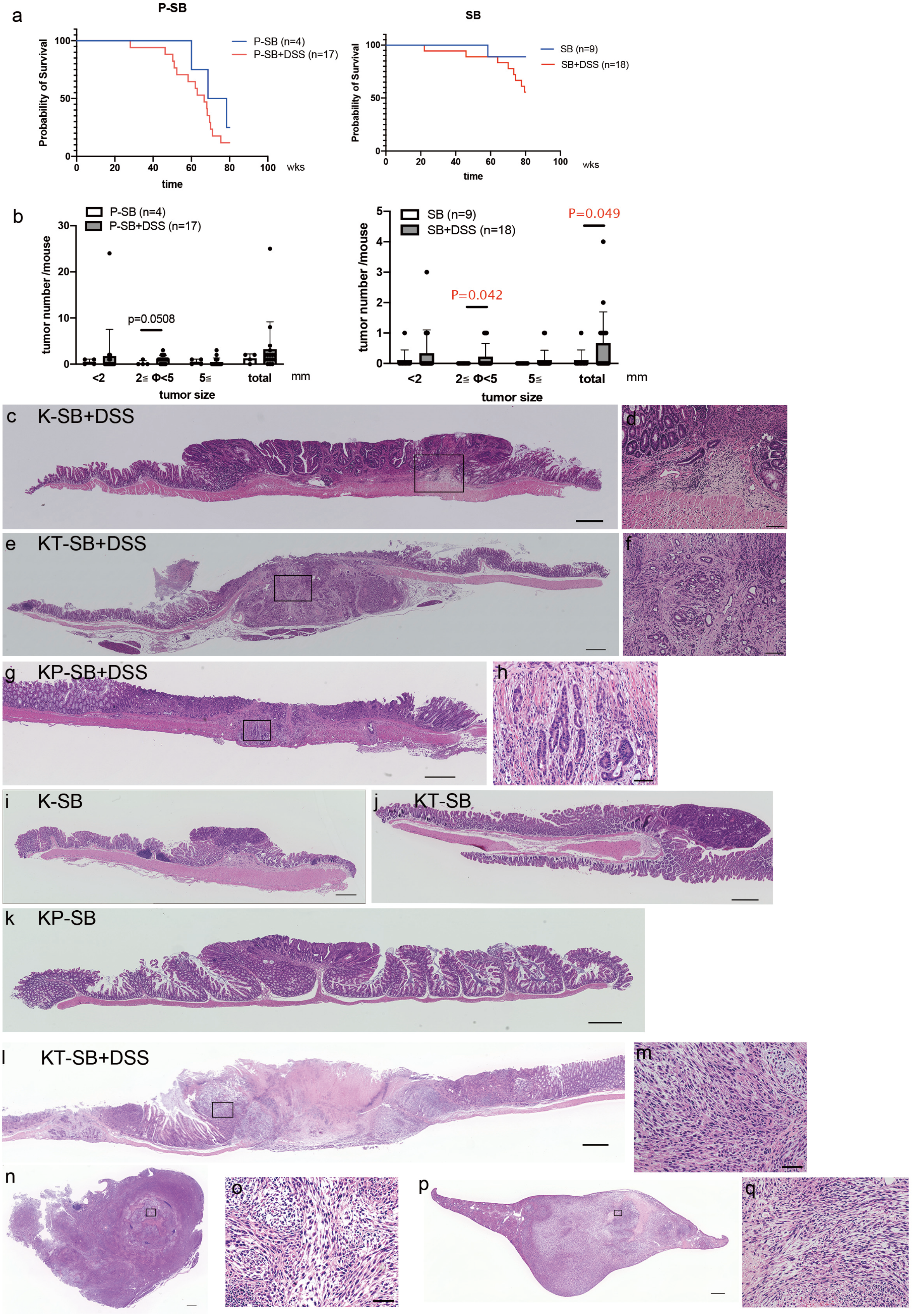

Supplementary Figure 1. Analyses of DSS-treated SB mice.

(a) Survivals for P-SB and SB mice, we stopped monitoring mice when they reached 80 wks of age and sent for necropsy. The average age of P-SB (71.8), P-SB+DSS (62.4 wks), SB (77.6) and SB+DSS (72.6) mice, Log-rank test. (b) The number of tumors developed in the colon of P-SB, P-SB+DSS, SB and SB+DSS mice. Two-sided t-test. Data are represented as mean values +/- SD. HE staining for representative colon tumors of K-SB+DSS mouse (c and d), KT-SB+DSS mouse (e and f), KP-SB+DSS mouse (g and h), K-SB mouse (i), KT-SB mouse (j), and KP-SB mouse (k). High-magnification images of the insets in (c), (e) and (f) are (d), (f) and (h), respectively. A KT-SB+DSS mouse developed a large invasive adenocarcinoma (l and m) which metastasized to the liver (n, o, p and q). High magnification images of the insets in (l), (n) and (p) are (m), (o) and (q), respectively. Scale bars, 0.5 mm (c, e, g, i, j, k, l, n and p), 100 μm (d and f) and 50 μm (h, m, o and q).

Supplementary Figure 2

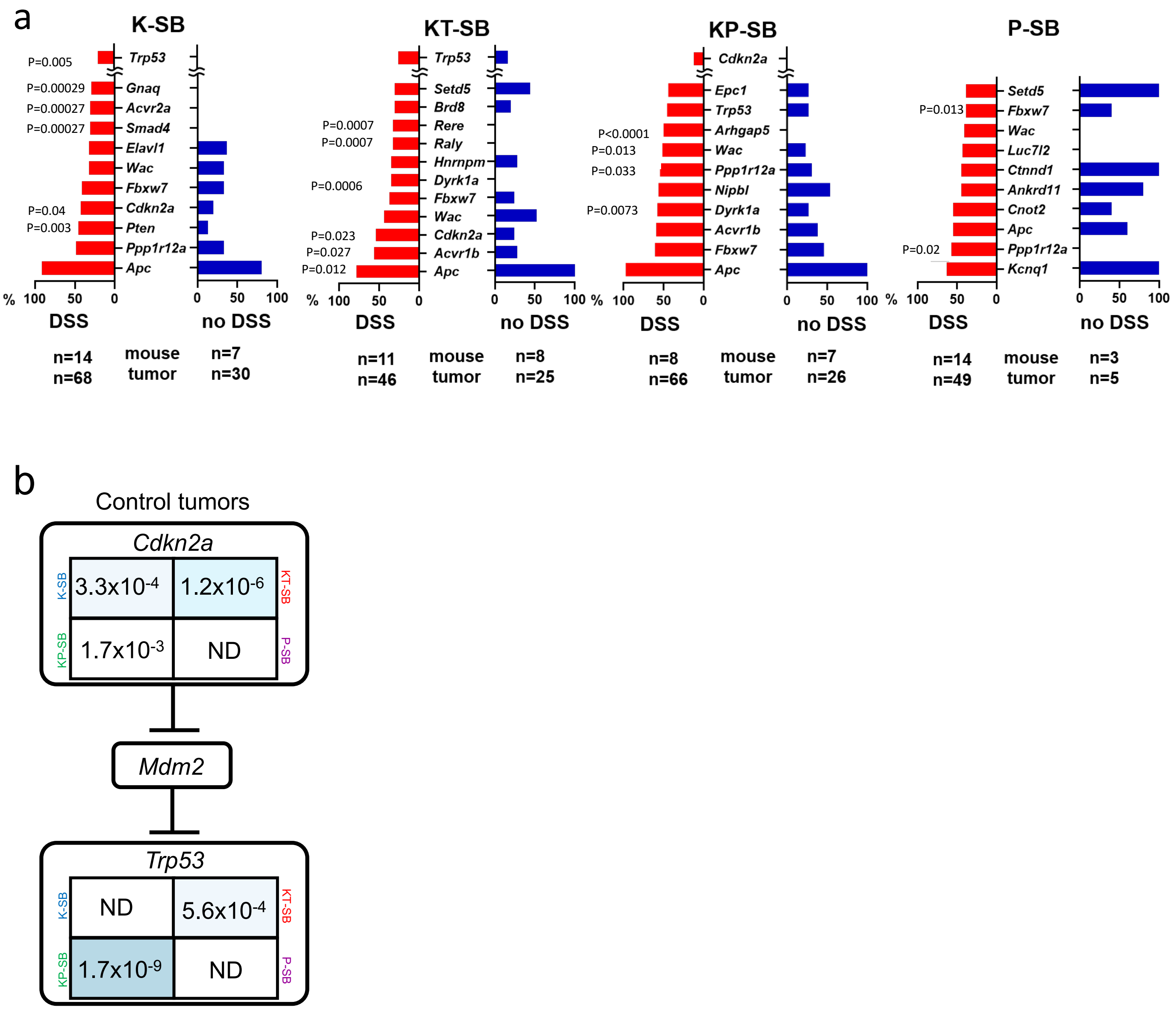

Supplementary Figure 2. Frequently mutated genes in inflammation-associated tumors.
(a) Top-10 genes identified from K-SB+DSS, KT-SB+DSS, KP-SB+DSS, and P-SB+DSS mice and the frequencies for the genes in their controls. Two-sided Fisher's exact test. (b) The p-values for *Cdkn2a* and *Trp53* in no DSS control tumors.

Supplementary Figure 3

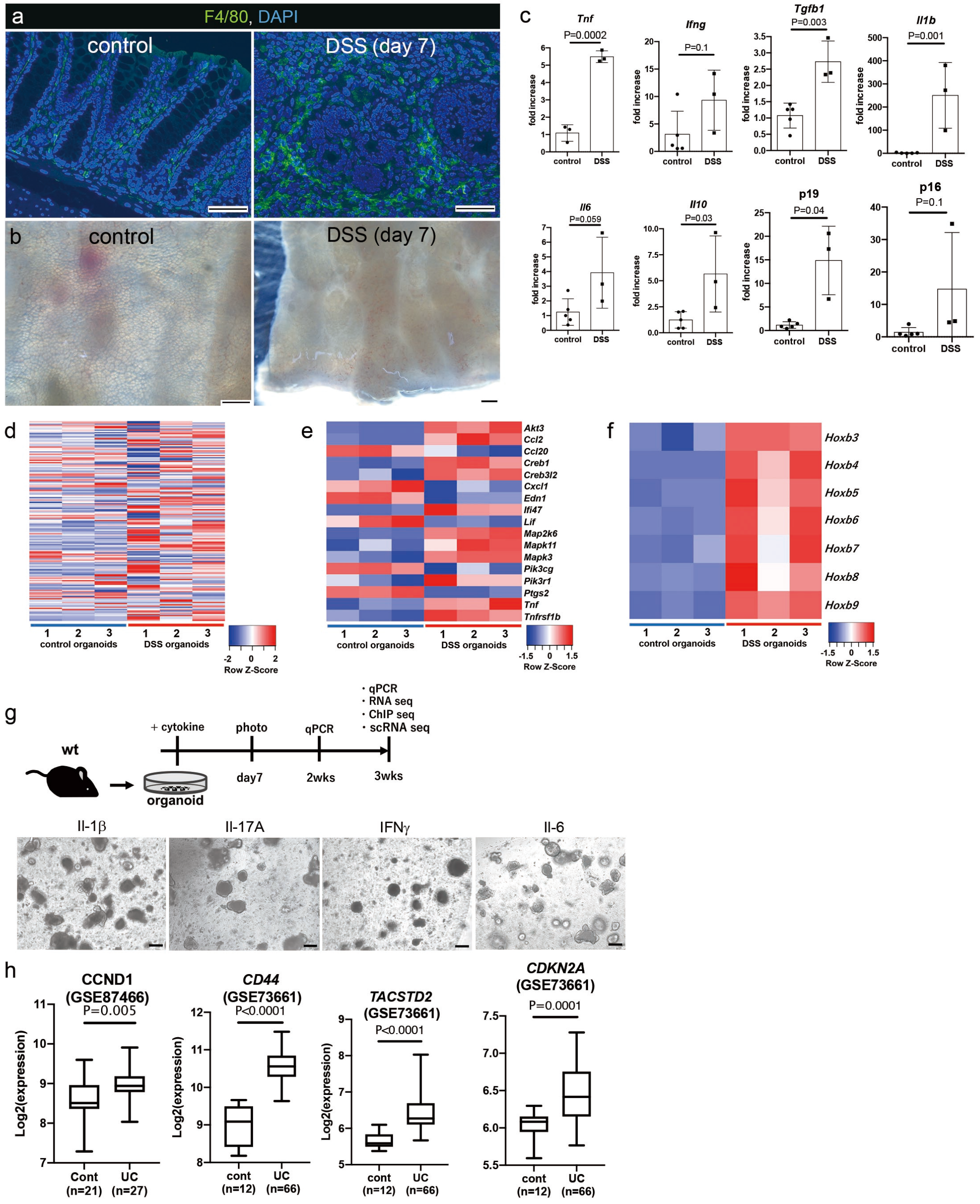

Supplementary Figure 3. Analyses of the DSS-treated mouse colon.

(a) Representative images of Immunofluorescent staining for the macrophage (F4/80) in the normal colonic mucosa (left) and the mucosa at day 7 from DSS treatment (right). Bars; 50 µm. At day 7 from DSS treatment, F4/80 (+) macrophages were accumulating near the damaged epithelial cells. (b) Macroscopic images of the normal mouse colonic mucosa (left) and the mucosa at day 7 from DSS treatment (right). Bars; 250 µm. (c) Quantitative PCR results for inflammatory cytokines and p16/p19 using the normal mouse colon tissues (n=5) (control) and the colon at day 7 from DSS treatment (DSS) (n=3). We observed increased expression of p16/p19*,* as well as inflammatory cytokines including TNFα, TGFβ and IFNγ. Two-sided t-test. The n indicates independent samples. Data are represented as mean values +/- SD. (d-f) Heatmaps showing the differentially expressed genes involved in senescence signaling (d), TNF signaling (e), and Hoxb cluster genes (f) between wt organoids and DSS organoids. (g) Photos of organoids with indicated cytokines. Addition of IFNγ caused organoid death. The morphology of organoids did not change when Il-6, Il-1β and Il-17 were added Bars; 250 µm. Three independent experiments were performed. (h) Box plots showing the RNA expression of colonic stem cell related genes in human colonic mucosa of the healthy control (cont) and UC patients (UC) derived from the GSE877466 and GSE73661 datasets. Two-sided t-test. The center lines indicate the mean value and whiskers indicate Min to Max.

Supplementary Figure 4

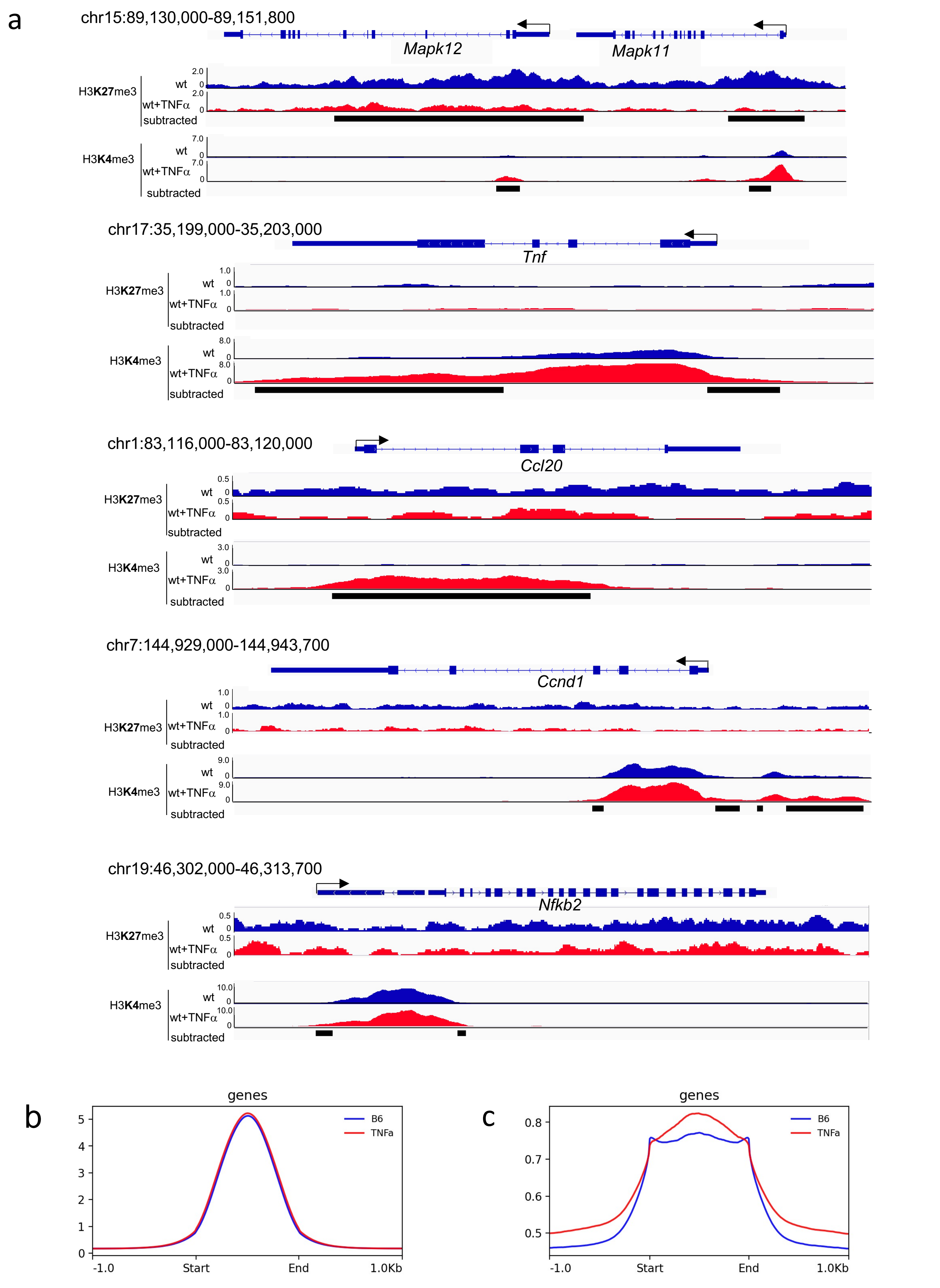

Supplementary Figure 4. Genes epigenetically deregulated by TNFα in mouse colonic organoids. (a) IGV images for ChIP-seq (H3K4me3 and H3K27me3) using wt organoids and wt+TNFα, for indicated genetic loci. (b, c) Pile up images for the intensity at transcription start sites for H3K4me3 (b), and H3K27me3 (c).

Supplementary Figure 5

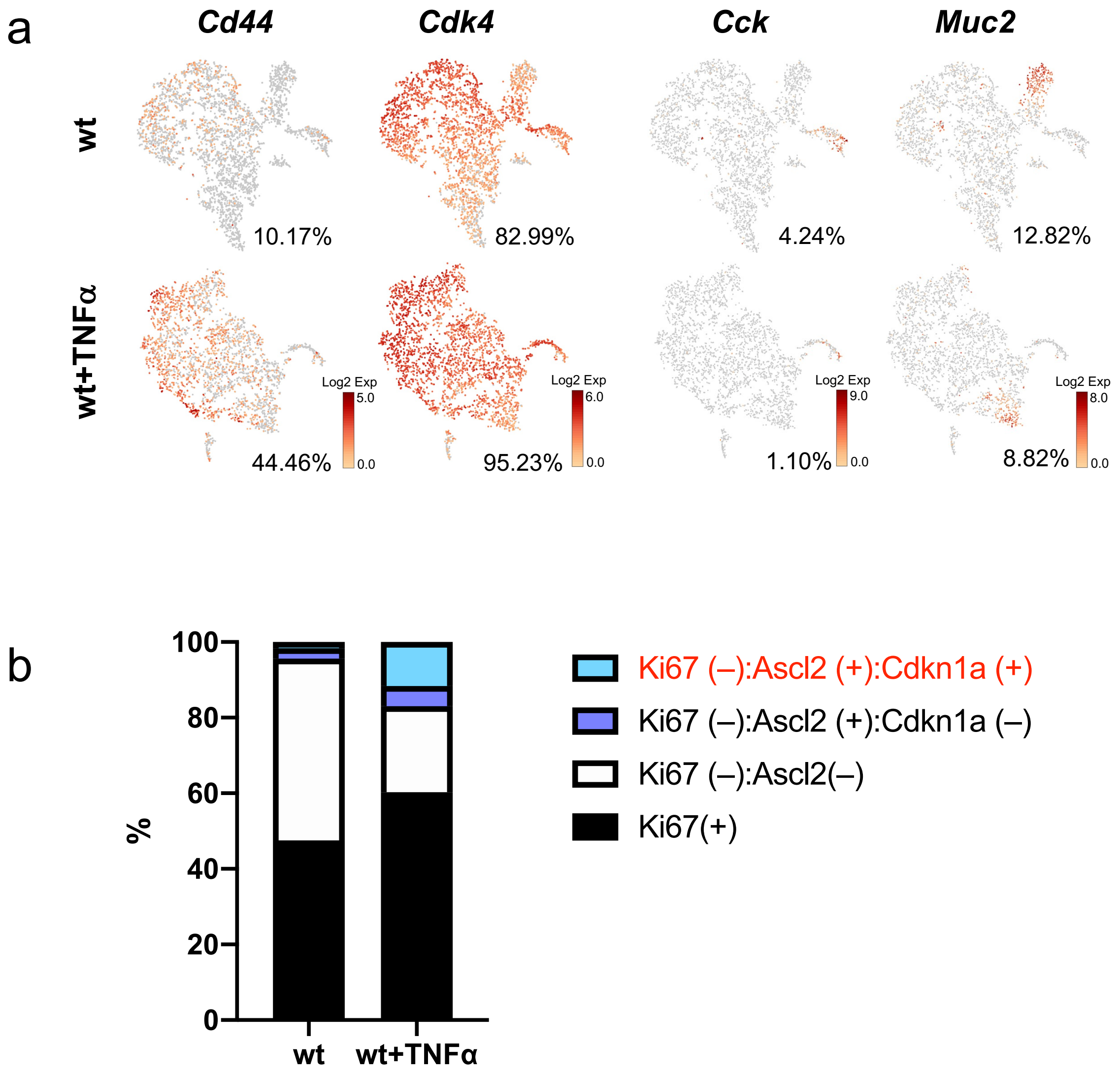

Supplementary Figure 5. Single cell RNA-seq using TNFα-treated wt organoids.
(a)T-SNE clustering analyses showing the cell population expressing indicated genes. (b) Bar graphs showing the proportion of double positive cells for *Ascl2*$^{+}$ and *Cdkn2a*$^{+}$ in all *Cdkn2a*$^{+}$ cells (left). Bar graphs showing the proportion of double positive cells for *Cd44*$^{+}$ and senescence^{+} in all senescence^{+} cells (middle). Senescence^{+} means cells expressing *Cdkn2a* and/or *Trp53*. Bar graphs showing the proportion of double positive cells for *Cd44*$^{+}$ and *Cdkn2a*$^{+}$ in all *Cdkn2a*$^{+}$ cells (right). (c) The proportion of *Cdkn2a*$^{+}$ cells, double positive cells for *Ascl2*$^{+}$ and *Cdkn2a*$^{+}$ and *Ascl2*$^{+}$ cells in *mki67* positive or negative cells.

Supplementary Figure 6

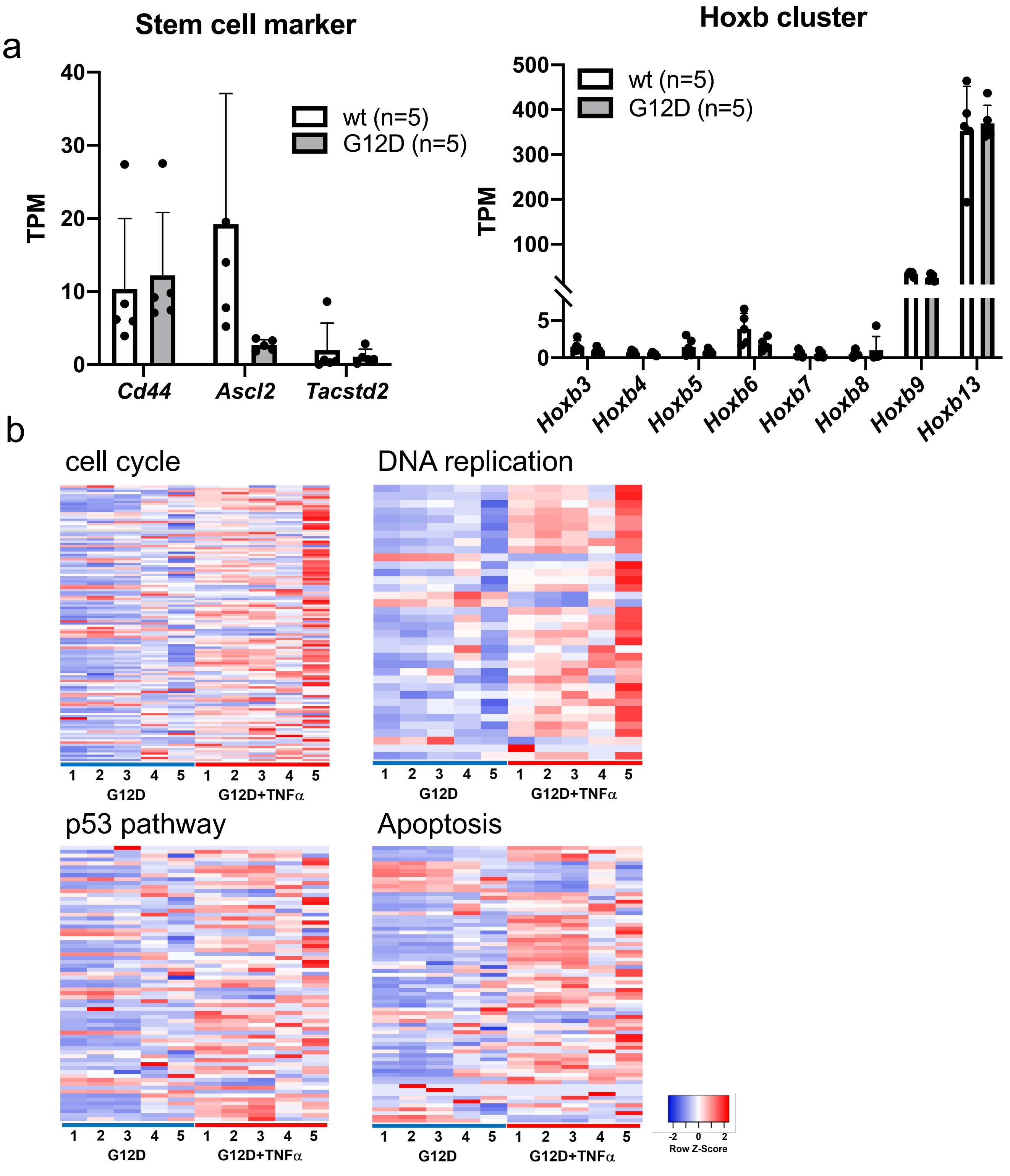

Supplementary Figure 6. Expression analyses using KrasG12D organoids in the absence or presence of TNFα.

(a) Bar graphs showing the expression (TPM values obtained by RNA-seq) of indicated genes in wt organoids and G12D organoids. Two-sided t-test. Data are represented as mean values +/- SD. The n indicates independent samples. (b) Heat maps showing the differentially expressed genes in “Cell cycle”, “DNA replication”, “p53 pathway” and “Apoptosis” signaling obtained by RNA-seq between KrasG12D organoids and KrasG12D organoids treated with TNFα for 3 wks.

Supplementary Figure 7

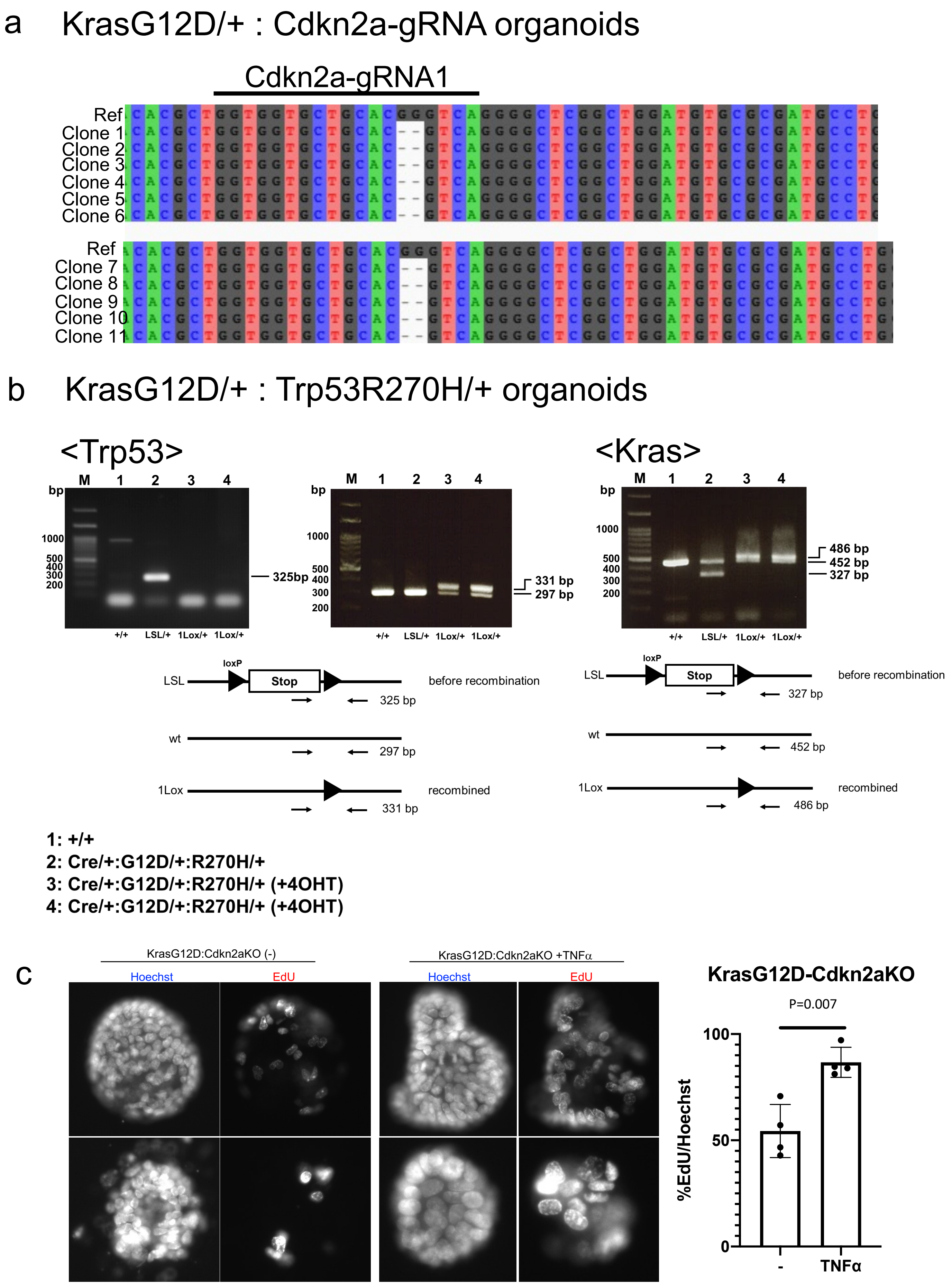

Supplementary Figure 7. The sequencing analyses for genomic locus targeted by *Cdkn2a*-gRNA.
(a) To confirm whether *Cdkn2a* was knocked out by CRISPR-Cas9 in KrasG12D/+ organoids used in Figure 4J, the target locus was PCR amplified and cloned into the vector. Eleven clones were sequenced and aligned to the reference sequence (top). Two-bp deletions were introduced in all clones, showing most of cells carried knockout alleles. (b) Recombination of loxP sequences by Cre recombinase was confirmed by PCR for *Trp53* and *Kras*. Three independent experiments were performed. (c) The fluorescence images of EdU positive cells (Red) and Hoechst positive cells (blue) in KrasG12D-Cdkn2a KO organoids with or without TNFα. Bar; 100 mm. The representative images of three independent experiments. The n indicates independent samples. The quantification of EdU positive cells / Hoechst positive cells from one representative experiment with four independent samples in each group. Two-sided t-test. Data are represented as mean values +/- SD.

Supplementary Figure 8

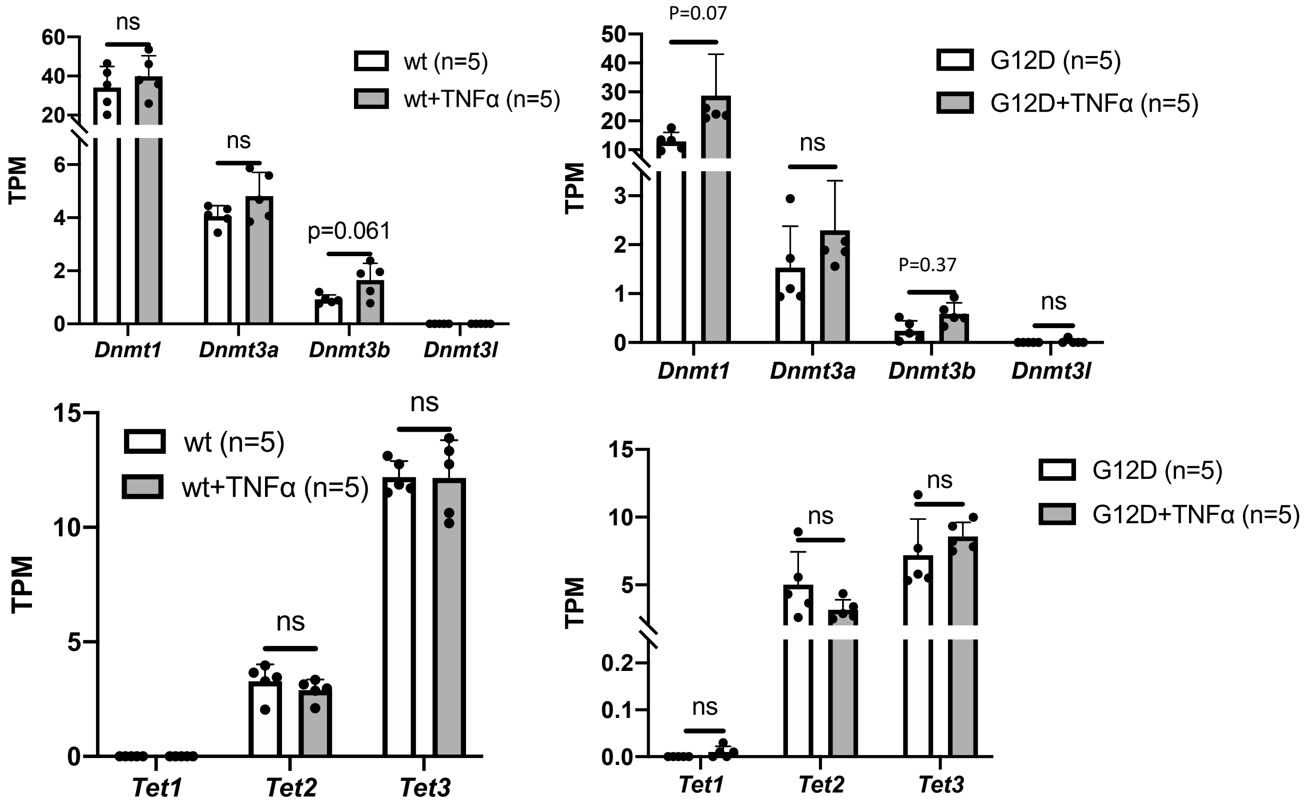

Supplementary Figure 8. **Long-term exposure of TNFα increased the level of DNA methylation in the *Cdkn2a* locus.**

Bar graphs showing the RNA expression of DNA methyltransferases and demethyltransferases in wt organoids or KrasG12D organoids in the absence or presence TNFα. The n indicates independent samples. Two-sided t-test. Data are represented as mean values +/- SD.

Supplementary Figure 9

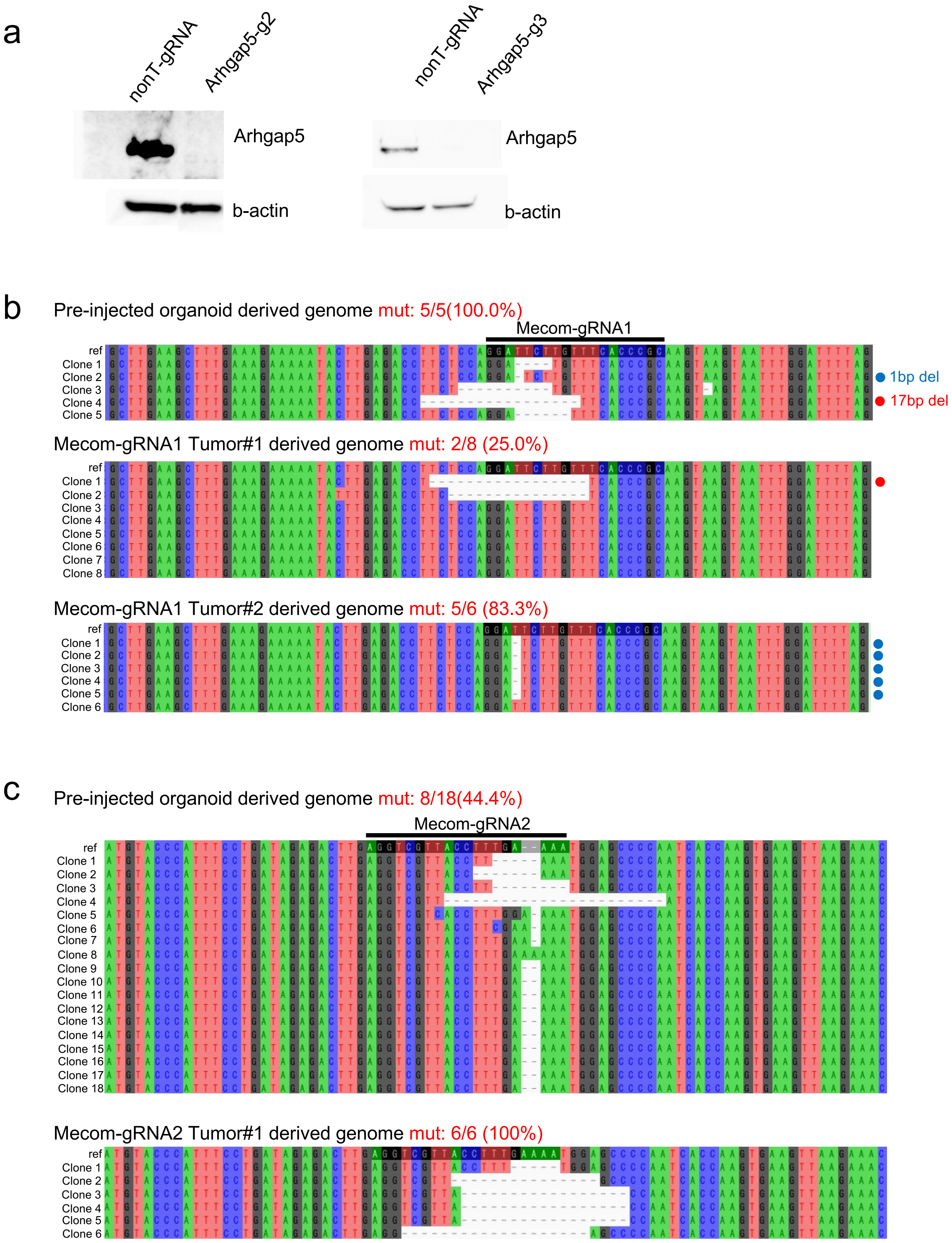

Supplementary Figure 9. **Confirmation of knockout for *Arhgap5* and *Mecom*.**
(a) Arhgap5 protein expression was detected by western blotting in AK-Cas9-nonT-gRNA (AK), AK-Cas9-Arhgap5-gRNA2 (Arhgap5-g2) and AK-Cas9-Arhgap5-gRNA3 (Arhgap5-g3). Three independent experiments were performed. (b) The *Mecom* gRNA1 targeting loci were sequenced to confirm efficient introduction of mutations in pre-injected organoids (up), subcutaneous tumor#1-derived genome (middle), and subcutaneous tumor#2 organoid-derived genome (bottom). Note that the same mutation pattern was observed between pre-injected organoids and tumor genome as shown by the red and blue circles. (c) The *Mecom* gRNA2 targeting loci were sequenced to confirm efficient introduction of mutations in pre-injected organoids (up), subcutaneous tumor#1-derived genome (bottom). These data showed that clones carrying frameshift mutations were enriched in the tumor genome.

Supplementary Tables

Supplementary Table 1. Sequences for qPCR primers

F primer sequence	R primer sequence	PrimerBank ID	target gene	species
CTGAACGGGAAGCTCACTGG	ATGCCTGCTTCACCACCTTC	-	Gapdh	mouse
AGGGCCGTGTGCATGACGTG	GCACCGGGCGGGAGAAGGTA	-	p16	mouse
CCTGGTGATGTCCGACCTG	CCATGAGCGCATCGCAATC	6671726a1	Cdkn1a	mouse
CGCAGGTTCTTGGTCACTGT	TGTTCACGAAAGCCAGAGCG	6753390a1	p19	mouse
GCCTCTCGTACATACAGACGC	CCAGTTCTGCTTTGGATCAGC	8394248a1	Ccl20	mouse
TTAAAAACCTGGATCGGAACCAA	GCATTAGCTTCAGATTTACGGGT	6755430a1	Ccl2	mouse
GCTCTACTCGTCTGGCTATGC	GTGGGTAATAGGAGGACGCC	21886813a1	Hoxb6	mouse
TGATACGCCTGAGTGGCTGTCT	CACAAGAGCAGTGAGCGCTGAA	-	Tgfb1	mouse
GGTGCCTATGTCTCAGCCTCTT	GCCATAGAACTGATGAGAGGGAG	-	Tnf	mouse
GCTCTTACTGACTGGCATGAG	CGCAGCTCTAGGAGCATGTG	6754318a1	Il10	mouse
TCTATACCACTTCACAAGTCGGA	GAATTGCCATTGCACAACTCTTT	13624310c2	Il6	mouse
ACAGCAAGGCGAAAAAGGATG	TGGTGGACCACTCGGATGA	145966741c2	Infg	mouse
GCAACTGTTCCTGAACTCAACT	ATCTTTTGGGGTCCGTCAACT	6680415a1	Il1b	mouse
GGAGATAGAGGACGACATTGGC	ACGCTCCACTACTGACAGGTCA	-	Inhba	mouse
GGCTGTATTCCCCTCCATCG	CCAGTTGGTAACAATGCCATGT	6671509a1	Actb	mouse
AACTCTTTCGGTCGTACCCC	GCGTGCTTGAGCTGAAGCTA	1162949a1	p16	mouse
GGAGCGAGATCCCTCCAAAAT	GGCTGTTGTCATACTTCTCATGG	378404907c1	GAPDH	human
ATGGAGCCTTCGGCTGACT	GTAACTATTCGGTGCGTTGGG	4502749a1	p16	human
GGGTTTTCGTGGTTCACATCC	CTAGACGCTGGCTCCTCAGTA	17738294a1	p14	human
TGCTGTACCAAGAGTTTGCTC	CGCACACAGACAACTTTTTCTTT	4759076a1	CCL20	human
GTGCTCCACTCCGGTCTAC	GTAACGTGTGTATGTCTGGCG	85543350c2	HOXB6	human

Supplementary Table 2. Sequences for qPCR primers to detect recombination in Kras and Trp53 alleles

primer	sequence
Kras-y116	TCCGAATTCAGTGACTACAGATG
Kras-y117	CTAGCCACCATGGCTTGAGT
Kras-y118	ATGTCTTTCCCCAGCACAGT
TP53-T035	CTTGGAGACATAGCCACACTG
Trp53-recombined	AGCCTGCCTAGCTTCCTCAGG

Supplementary Table 3. gRNA sequences

gRNA name	target sequence	species
Cdkn2a-gRNA1	GGTGGTGCTGCACGGGTCA	mouse
Cdkn2a-gRNA2	CCGGGCGGGAGAAGGTAGT	mouse
Cdkn2a-gRNA3	CAGCTCTTCTGCTCAACTA	mouse
Arhgap5-gRNA2	ACATTTGTACCGATCAGCT	mouse
Arhgap5-gRNA3	GTTCAATCTCTTCTAGATC	mouse
non-targetT	TATTACTGATATTGGTGGG	mouse
Mecom-gRNA1	GCGGGTGAAACAAGAATCC	mouse
Mecom-gRNA2	AGGTCGTTACCTTTGAAAA	mouse

Source data

Supplementary Fig 7b

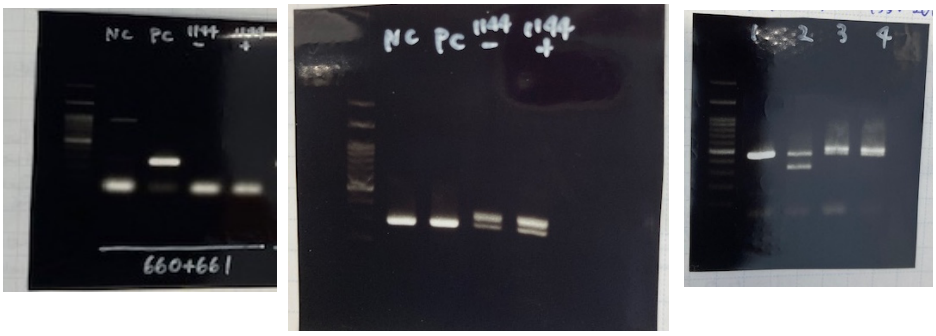

Supp Fig 9

nonT-gRNA Arhgap5-g2

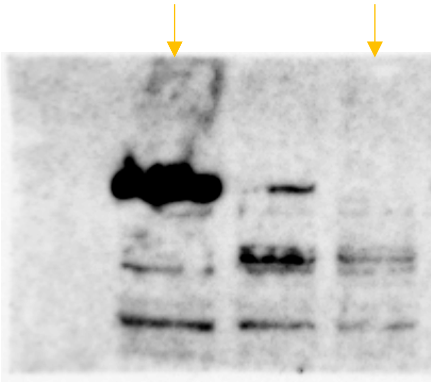

Supp Fig 9

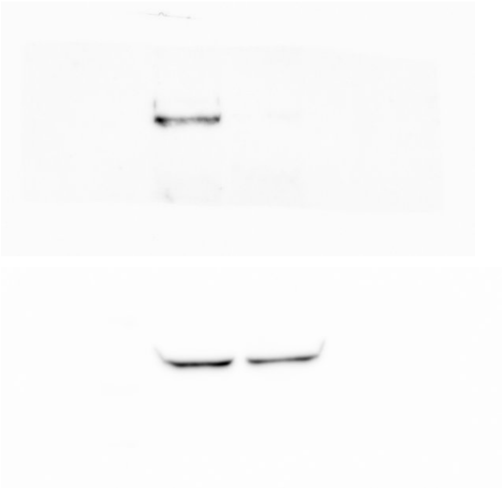

nonT-gRNA Arhgap5-g2

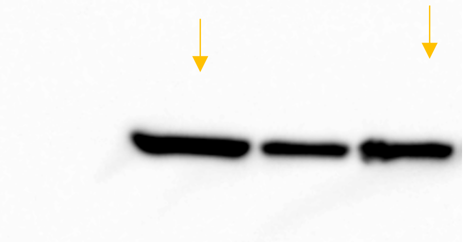

Supplement: Supplementary file 1 — Supplementary Information [file 41467_2023_42228_MOESM1_ESM.pdf]
