# Supplementary material for: Sleeping Beauty transposon mutagenesis identified genes and pathways involved in inflammation-associated colon tumor development
Source: Nat Commun. 2023 Oct 16;14:6514. doi: 10.1038/s41467-023-42228-z (PMC10579371; doi:10.1038/s41467-023-42228-z)
Supplement: Supplementary file 3 — Reporting Summary [file 41467_2023_42228_MOESM3_ESM.pdf]

Corresponding author(s): Haruna Takeda
Last updated by author(s): 2023/9/8

Reporting Summary

Nature Portfolio wishes to improve the reproducibility of the work that we publish. This form provides structure for consistency and transparency in reporting. For further information on Nature Portfolio policies, see our Editorial Policies and the Editorial Policy Checklist.

Statistics

For all statistical analyses, confirm that the following items are present in the figure legend, table legend, main text, or Methods section.

n/a	Confirmed	
☐	☒	The exact sample size (*n*) for each experimental group/condition, given as a discrete number and unit of measurement
☐	☒	A statement on whether measurements were taken from distinct samples or whether the same sample was measured repeatedly
☐	☒	The statistical test(s) used AND whether they are one- or two-sided<br>*Only common tests should be described solely by name; describe more complex techniques in the Methods section.*
☒	☐	A description of all covariates tested
☒	☐	A description of any assumptions or corrections, such as tests of normality and adjustment for multiple comparisons
☐	☒	A full description of the statistical parameters including central tendency (e.g. means) or other basic estimates (e.g. regression coefficient) AND variation (e.g. standard deviation) or associated estimates of uncertainty (e.g. confidence intervals)
☐	☒	For null hypothesis testing, the test statistic (e.g. *F*, *t*, *r*) with confidence intervals, effect sizes, degrees of freedom and *P* value noted<br>*Give P values as exact values whenever suitable.*
☒	☐	For Bayesian analysis, information on the choice of priors and Markov chain Monte Carlo settings
☒	☐	For hierarchical and complex designs, identification of the appropriate level for tests and full reporting of outcomes
☒	☐	Estimates of effect sizes (e.g. Cohen's *d*, Pearson's *r*), indicating how they were calculated

Our web collection on statistics for biologists contains articles on many of the points above.

Software and code

Policy information about availability of computer code

Data collection	All the software for data collection was described in the manuscript.
Data analysis	Custom codes for the Sleeping Beauty screening analysis were deposited in https://github.com/ni6o6/sb.

For manuscripts utilizing custom algorithms or software that are central to the research but not yet described in published literature, software must be made available to editors and reviewers. We strongly encourage code deposition in a community repository (e.g. GitHub). See the Nature Portfolio guidelines for submitting code & software for further information.

Data

Policy information about availability of data

All manuscripts must include a data availability statement. This statement should provide the following information, where applicable:

- Accession codes, unique identifiers, or web links for publicly available datasets
- A description of any restrictions on data availability
- For clinical datasets or third party data, please ensure that the statement adheres to our policy

The RNA-seq data and the ChIP-seq data generated in this study have been deposited in the Gene Expression Omnibus (GEO) database under accession code GSE217170 [https://www.ncbi.nlm.nih.gov/geo/query/acc.cgi?acc=GSE217170], and GSE221326 [https://www.ncbi.nlm.nih.gov/geo/query/acc.cgi?acc=GSE221326], respectively. The scRNA-seq data was deposited in the DDBJ under accession code SAMD00641686 [https://ddbj.nig.ac.jp/resource/biosample/SAMD00641686], SAMD00641687 [https://ddbj.nig.ac.jp/resource/biosample/SAMD00641687].

We use OncoKB data base [https://www.oncokb.org/cancerGenes], NCBI38/mm10 [https://www.ncbi.nlm.nih.gov/datasets/genome/GCF_000001635.20/] for analyses.

Research involving human participants, their data, or biological material

Policy information about studies with human participants or human data. See also policy information about sex, gender (identity/presentation), and sexual orientation and race, ethnicity and racism.

Reporting on sex and gender	We reported the gender of the person from whom the sample originated.
Reporting on race, ethnicity, or other socially relevant groupings	We reported the race of the person from whom the sample originated.
Population characteristics	We used two samples derived from 2 Japanese male patients.
Recruitment	Written informed consent was obtained in advance for participants.
Ethics oversight	No compensation was paid to the participant.

Note that full information on the approval of the study protocol must also be provided in the manuscript.

Field-specific reporting

Please select the one below that is the best fit for your research. If you are not sure, read the appropriate sections before making your selection.

- [x] Life sciences
- [ ] Behavioural & social sciences
- [ ] Ecological, evolutionary & environmental sciences

For a reference copy of the document with all sections, see nature.com/documents/nr-reporting-summary-flat.pdf

Life sciences study design

All studies must disclose on these points even when the disclosure is negative.

Sample size	No statistical method was used to predetermine sample size, but sample size was set to obtain the maximum results with the minimum number of samples to perform statistical analyses.
Data exclusions	No data were excluded.
Replication	Experimental results were obtained by three independent experiments, and only those with confirmed reproducibility are included.
Randomization	Mice were randomly divided into DSS-treated and untreated groups.
Blinding	The investigators were not blinded to allocation during experiments and outcome assessment.

Reporting for specific materials, systems and methods

We require information from authors about some types of materials, experimental systems and methods used in many studies. Here, indicate whether each material, system or method listed is relevant to your study. If you are not sure if a list item applies to your research, read the appropriate section before selecting a response.

Materials & experimental systems

n/a	Involved in the study	
[ ]	[x]	Antibodies
[x]	[ ]	Eukaryotic cell lines
[x]	[ ]	Palaeontology and archaeology
[ ]	[x]	Animals and other organisms
[x]	[ ]	Clinical data
[x]	[ ]	Dual use research of concern
[x]	[ ]	Plants

Methods

n/a	Involved in the study	
[ ]	[x]	ChIP-seq
[x]	[ ]	Flow cytometry
[x]	[ ]	MRI-based neuroimaging

Antibodies

Antibodies used	anti-H3K4me3 Ab (Millipore, #05-745R, clone 15-10-E4, rabbit monoclonal, 3 µl)<br>anti-H3K27me3 Ab (Cell Signaling Technology, #9733S, clone C36B11, rabbit monoclonal, 5 µl)

Validation	Broad species cross-reactivity of anti-H3K4me3 Ab (Millipore #05-745R) is expected due to sequence homology. This antibody is proven in ChIP-seq to detect H3K4me3 according to the manufacture's web site (https://www.merckmillipore.com/JP/ja/product/Anti-trimethyl-Histone-H3-Lys4-Antibody-clone-15-10C-E4-rabbit-monoclonal,MM_NF-05-745R?ReferrerURL=https%3A%2F%2Fwww.google.com%2F#overview) and by multiple previous studies using mouse genome (e.g. Shen et al, Nature, 488:116-120, 2012).<br>Anti-H3K27me3 Ab (Cell Signaling Technology #9733S) reacts with mouse Tri-Methyl-Histone H3 (Lys27) and is proven in ChIP-seq according to the manufacture's web site (https://www.cellsignal.jp/products/primary-antibodies/tri-methyl-histone-h3-lys27-c36b11-rabbit-mab/9733). In addition, the reactivities of these antibodies has been validated by multiple previous studies (e.g. Wu et al, Nat Commun, 470:13, 2022).

Animals and other research organisms

Policy information about studies involving animals; ARRIVE guidelines recommended for reporting animal research, and Sex and Gender in Research

Laboratory animals	General laboratory mice were used. The genetic background of mice used in each experimental is reported in the Source Data file.
Wild animals	The study did not involve wild animals.
Reporting on sex	The age of each mouse is reported in the Source Data file.
Field-collected samples	The study did not involve the samples collected from the field.
Ethics oversight	All protocols for animal experiments were reviewed and approved by the institutional animal care and use committee of National Cancer Center (Study number: T19-006-M07). The study protocol for experiments on human-derived samples was approved by the Ethics Committees of the National Cancer Center (Study number: 2020-393, 2008-097). Written informed consent was obtained in advance for participants, and no compensation was paid.

Note that full information on the approval of the study protocol must also be provided in the manuscript.

Plants

Seed stocks	n/a
Novel plant genotypes	n/a
Authentication	n/a

ChIP-seq

Data deposition

- [x] Confirm that both raw and final processed data have been deposited in a public database such as GEO.
- [x] Confirm that you have deposited or provided access to graph files (e.g. BED files) for the called peaks.

Data access links<br>*May remain private before publication.*	All high-throughput sequencing data generated in this study are accessible at GEO via GEO accession number GSE221326 (https://www.ncbi.nlm.nih.gov/geo/query/acc.cgi?acc=GSE221326).
Files in database submission	B6_In_DKDL22H000001-1a-AK2451_H72FFDSX3_L3_1.fq.gz<br>B6_In_DKDL22H000001-1a-AK2451_H72FFDSX3_L3_2.fq.gz<br>B6_K4_R1.fastq.gz<br>B6_K4_R2.fastq.gz<br>B6_K27_R1.fastq.gz, B6_K27_R2.fastq.gz<br>TNFa_IN_R1.fastq.gz<br>TNFa_IN_R2.fastq.gz<br>TNFa_K4_R1.fastq.gz<br>TNFa_K4_R2.fastq.gz<br>TNFa_K27_R1.fastq.gz<br>TNFa_K27_R2.fastq.gz<br>B6_K4_bwa.Q25_fixmate_markdup_SpikeIn.white_peaks.narrowPeak<br>B6_K27_bwa.Q25_fixmate_markdup_SpikeIn_macs2_broad0.01_peaks.broadPeak<br>TNFa_K4_bwa.Q25_fixmate_markdup_SpikeIn.white_peaks.narrowPeak<br>TNFa_K27_bwa.Q25_fixmate_markdup_macs2_broad0.01_peaks.broadPeak
Genome browser session<br>(e.g. UCSC)	Available at GEO

Methodology

Replicates	Single
Sequencing depth	Name: Total_reads, Unique_reads, Reads_length, Reads_type<br>B6_Input: 34358934, 34186140, 150, PE<br>B6_H3K4me3: 46148719, 45815354, 150, PE<br>B6_H3K27me3: 102912657, 102172818, 150, PE<br>TNFa_Input: 60183325, 59926704, 150, PE<br>TNFa_H3K4me3: 76022679, 75701964, 150, PE<br>TNFa_H3K27me3: 102830474, 102541252, 150, PE
Antibodies	anti-H3K4me3 Ab (clone 15-10C-E4, Millipore #05-745R) anti-H3K27me3 Ab (C36B11, Cell Signaling Technology #9733S),
Peak calling parameters	H3K4me3: Macs2: -f --SPMR -p 1e9<br>H3K27me3: Macs2: -f --SPMR -broad --broad-cutoff 0.01
Data quality	H3K4me3 peaks were called with q-value threshold of 10^-7.<br>H3K27me3 peaks were called with q-value threshold of 10^-2.
Software	fastp (version 0.20.0)<br>bwa-mem (version 2.2)<br>SAMtools (version 1.11)<br>MACS2 (version 2.7.1)<br>deeptools (version 3.5.1)<br>bedtools (version 2.30.0)
