# Supplementary material for: Sleeping Beauty transposon mutagenesis identified genes and pathways involved in inflammation-associated colon tumor development
Source: Nat Commun. 2023 Oct 16;14:6514. doi: 10.1038/s41467-023-42228-z (PMC10579371; doi:10.1038/s41467-023-42228-z)
Supplement: Supplementary file 4 — Description of Additional Supplementary Files [file 41467_2023_42228_MOESM4_ESM.pdf]

Supplementary Data

Supplementary Data 1. 1,459 CCDGs identified from SB screens.

Supplementary Data 2. Overlapping genes identified from the previous SB screens and the SB screens in the present study.

Supplementary Data 3. Inflammation-associated tumor-related genes.

Supplementary Data 4. Genes identified from ChIP-seq.

Supplementary Data 5. Pathway analyses for genes identified from ChIP-seq using wt organoids and TNFα-treated wt organoids.

Supplementary Data 6. Genes identified from ChIP-seq and RNA-seq.

Supplementary Data 7. Overlapping genes between SB screens and TCGA datasets.
